# cGMP Analogues with Opposing Actions on CNG Channels Selectively Modulate Rod or Cone Photoreceptor Function

**DOI:** 10.3390/pharmaceutics14102102

**Published:** 2022-10-01

**Authors:** Sophie Wucherpfennig, Wadood Haq, Valerie Popp, Sandeep Kesh, Soumyaparna Das, Christian Melle, Andreas Rentsch, Frank Schwede, François Paquet-Durand, Vasilica Nache

**Affiliations:** 1 Institute of Physiology II, University Hospital Jena, Friedrich Schiller University Jena, 07743 Jena, Germany; 2 Neuroretinal Electrophysiology and Imaging, Institute for Ophthalmic Research, University of Tübingen, 72076 Tübingen, Germany; 3 Cell Death Mechanism Group, Institute for Ophthalmic Research, University of Tübingen, 72076 Tübingen, Germany; 4 Biomolecular Photonics Group, University Hospital Jena, Friedrich Schiller University Jena, 07743 Jena, Germany; 5 Biolog Life Science Institute GmbH & Co. KG, 28199 Bremen, Germany

**Keywords:** CNG channels, retinal degeneration, target selectivity, *Retinitis pigmentosa*, ion channels, cGMP analogues, drug therapy

## Abstract

The vertebrate retina harbors rod and cone photoreceptors. Human vision critically depends on cone photoreceptor function. In the phototransduction cascade, cGMP activates distinct rod and cone isoforms of the cyclic nucleotide-gated (CNG) channel. Excessive cGMP levels initiate a pathophysiological rollercoaster, which starts with CNG channel over-activation, typically in rod photoreceptors. This triggers cell death of rods first, and then cones, and is the root cause of many blinding retinal diseases, including *Retinitis pigmentosa*. While targeting of CNG channels has been proposed for therapeutic purposes, thus far, it has not been possible to inhibit rod CNG channels without compromising cone function. Here, we present a novel strategy, based on cGMP analogues with opposing actions on CNG channels, which enables the selective modulation of either rod or cone photoreceptor activity. The combined treatment with the weak rod-selective CNG-channel inhibitor (Rp-8-Br-PET-cGMPS) and the cone-selective CNG-channel activator (8-pCPT-cGMP) essentially normalized rod CNG-channel function while preserving cone functionality at physiological and pathological cGMP levels. Hence, combinations of cGMP analogues with desired properties may elegantly address the isoform-specificity problem in future pharmacological therapies. Moreover, this strategy may allow for improvements in visual performance in certain light environments.

## 1. Introduction

The phototransduction cascade takes place in the outer segments of photoreceptors and undergoes similar steps in rod and cone photoreceptors. To work under dim-light and bright-light conditions, respectively, rods and cones possess a complex photoreceptor-type-specific cluster of proteins that is responsible for their distinct functional properties [1,2]. A key player in the phototransduction cascade is the cyclic nucleotide-gated (CNG) channel. In the absence of light, these channels are kept open by high cGMP levels, estimated to be ~3–5 µM in rod photoreceptors, and trigger the so-called “dark current” [3]. Upon illumination, rhodopsin activation sets in motion a cascade of events leading to a decrease in the cGMP concentration. As a result, CNG channels close, causing membrane hyperpolarization and an abrupt decrease in synaptic transmitter release [4,5]. 

Rod and cone CNG channels differ not only in their subunit composition but also in their functional characteristics [5]. They consist typically of CNGA- and CNGB-type subunits [6,7,8]. The rod CNGA1 and cone CNGA3 subunits confer the main channel properties and can form functional homotetrameric channels in heterologous expression systems. The modulatory subunits, rod CNGB1a and cone CNGB3, are not able to form functional homotetrameric channels, but play a decisive role in membrane trafficking and channel modulation through Ca^2+^/calmodulin [9,10,11,12]. Moreover, differences between the CNG-channel isoforms were reported regarding their ion permeation, ligand sensitivity, and gating kinetics of activation and deactivation [5]. 

Inherited retinal degenerative diseases are typically caused by the degeneration of either rod photoreceptors, as in the case of *Retinitis pigmentosa* (RP) [13,14], or cone photoreceptors, as in the case of *achromatopsia* or Stargardt disease [15,16]. In RP, the primary loss of rod photoreceptors is followed by a secondary degeneration of cones, leading to neuronal and vascular remodeling of the retina, severe visual impairment, and ultimately to blindness, with no efficient cure so far [17,18]. To date, mutations in more than 300 genes have been identified as causes of retinal degeneration diseases (Retina Information Network; http://www.sph.uth.tmc.edu/RetNet/, accessed on 23 September 2022). As recently shown by whole-exome sequencing analysis, the largest number of mutated genes are related to impaired structure and function of ion channels and their modulators [19]. This includes reduced folding flexibility of the channel proteins, faulty trafficking to the plasma membrane, and altered neurotransmitter regulation.

Due to its high genetic heterogeneity, the development of a single therapy for all forms of this disease is a challenge [20]. In many RP forms, an increased level of cGMP was observed in affected photoreceptors [17,21,22,23], yet the pathway by which the cGMP-homeostasis imbalance leads to photoreceptor death is still unclear. The main cellular targets for elevated cGMP are the CNG channels and cGMP-dependent protein kinase (PKG) [24]. In a study on the *rd1*-mouse model for RP, lacking the phosphodiesterase PDE6 β-subunit, degeneration of rods was attributed to the massive opening of CNG channels and the subsequent Ca^2+^ increase [25]. Moreover, when membrane incorporation of rod CNG channels is hindered by deletion of the CNGB1a subunit, retinal degeneration in the *rd1* mouse was significantly delayed [26]. Similarly, exacerbated activation of PKG has been shown to cause cell death in several cell types and RP-animal models [27,28,29]. Thus, cGMP signaling has become a promising target for pharmaceutical approaches [30]. 

Several ongoing strategies aim to delay rod death so as to prevent the secondary loss of cone photoreceptors [31,32]. Recently, photoreceptor degeneration was successfully delayed by application of inhibitory Rp-configurated phosphorothioate-modified cGMP analogues. In particular, Rp-8-Br-PET-cGMPS (CN03) robustly protected rod photoreceptors and preserved cone-photoreceptor function in three different RP-mouse models [30]. The effectiveness of this treatment was attributed to the simultaneous inhibition of both high-cGMP targets: the “Rp” modification to cGMP typically inhibits PKG [33], while the “β-phenyl-1,N^2^-etheno” modification (PET) inhibits CNG channels [34]. However, the direct effect of this and other cGMP analogues on rod and cone CNG-channel isoforms, and whether they might be channel-isoform-selective, is unknown. While indiscriminate inhibition of CNG channels might prevent rod degeneration, it would also result in loss of cone-mediated vision. Therefore, a pharmaceutical treatment should target only the desired photoreceptor, without hindering the function of the respective other photoreceptor type. 

The aim of this study was to identify cGMP analogues that could selectively target the CNG-channel isoform of interest, such that, in RP, only the pathologically increased activity of rod channels would be inhibited. While we were unable to identify a selective rod-channel inhibitor, we found a selective cone-channel activator. By combining a general CNG-channel inhibitor with a cone-channel activator, we were able to inhibit rod channels and preserve cone-channel function, at pathological, RP-like cGMP concentrations. The effectiveness of this approach was tested first on CNG channels expressed in *Xenopus laevis* oocytes and further confirmed by micro-electroretinography (µERG) recordings on wild-type mouse retinas.

## 2. Materials and Methods

### 2.1. Animals

The procedures regarding the *Xenopus laevis* frogs were approved by the animal ethics committee of the Friedrich Schiller University Jena (UKJ-18-008 from 09 May 2018). The respective protocols were performed in accordance with the approved guidelines. Extreme efforts were made to reduce the stress and to keep the number of frogs to a minimum.

Further animal experiments were performed using adult postnatal-day (P) 25–30 C57BL/6J mice (*n* = 12). Animals were housed under standard 12 h white cyclic lighting, had free access to food, water, and playgrounds, and were used irrespective of gender. Animal protocols compliant with §4 of the German law on animal protection were reviewed and approved by the Tübingen University committee on animal protection, and registered on 30 March 2022 under the No. AK05/22M. Moreover, experiments were performed in accordance with the ARVO statement for the use of animals in ophthalmic and visual research. All efforts were made to minimize the number of mice used and their suffering.

### 2.2. Molecular Biology and Heterologous Expression of Photoreceptor CNG Channels in Xenopus laevis Oocytes

For heterologous expression in *Xenopus laevis* oocytes, bovine CNGA1 (NM_ 174278.2) and CNGB1a subunits (NM_181019.2) from rod photoreceptors and human CNGA3 (NM_001298.2) and CNGB3 subunits (NM_019098.4) from cone photoreceptors were subcloned into the pGEMHE vector [35]. The cDNA encoding for the human-cone CNGB3 subunit was kindly provided by M. Varnum (Washington State University, Department of Veterinary and Comparative Anatomy, Pharmacology and Physiology, Pullman, Pullman, WA, USA), and for the bovine CNGB1a subunit, by W. Zagotta (University of Washington, Department of Physiology and Biophysics, Seattle, WA, USA).

Oocytes were surgically removed from adult females of *Xenopus laevis* under anesthesia with 0.1% tricaine, pH = 7.1 (MS-222, Pharmaq LTD Fordingbridge Hampshire SP6 1PA, UK), and were then incubated for 105 min in Ca^2+^-free solution (in mM: 82.5 NaCl, 2 KCl, 1 MgCl_2_, and 5 HEPES, pH 7.4) containing 3 mg/mL collagenase A (Roche Diagnostics, Mannheim, Germany). Oocytes of stage IV and V were manually defolliculated and each oocyte was injected with 50–100 ng of cRNA encoding for the respective photoreceptor CNG channels. The vitelline membrane was manually removed from the oocyte before the electrophysiological experiments. For efficient generation of heterotetrameric channels, the ratio of CNGA3 cRNA to CNGB3 cRNA was 1:2.5 [7] and that of CNGA1 cRNA to CNGB1a cRNA was 1:4 [36,37]. After the cRNA injection, the oocytes were kept at 18 °C for 2 to 7 days in a solution containing (in mM) 84 NaCl, 1 KCl, 2.4 NaHCO_3_, 0.82 MgSO_4_, 0.41 CaCl_2_, 0.33 Ca(NO_3_)_2_, and 7.5 Tris, with pH 7.4.

### 2.3. Electrophysiology

Patch-clamp recordings were performed on inside-out patches from *Xenopus laevis* oocytes expressing heterotetrameric rod and cone CNG channels. Recordings were made at room temperature using an Axopatch 200B patch-clamp amplifier (Axon Instruments, Foster City, CA, USA). Electrophysiology was controlled by the PatchMaster software (HEKA Elektronik Dr. Schulze GmbH, Lambrecht, Germany). The sampling rate was 5 kHz, and the filter implemented in the amplifier was set to 2 kHz. From a holding potential of 0 mV, currents were elicited by voltage steps to −100 mV, then to +100 mV, and back to 0 mV. When mentioned, voltage steps to −35 mV—the physiological voltage under dark conditions in photoreceptors—were applied.

Intracellular and extracellular solutions contained 140 mM NaCl, 5 mM KCl, 1 mM EGTA, and 10 mM HEPES (pH 7.4). The cyclic nucleotides, cAMP (Merck KGaA, Darmstadt, Germany), cGMP, and cGMP analogues (Biolog LSI GmbH & Co. KG, Bremen, Germany) were added to intracellular solutions as indicated. The concentrations of the respective dilutions were verified by UV spectroscopy (Thermo NanoDrop 2000c, Bremen, Germany). The L-*cis*-diltiazem solutions (100 µM, Abcam, Berlin, Germany) were prepared from stock solutions (10 mM) shortly before the measurements.

The patch pipettes were pulled from borosilicate glass tubing (outer diameter 2.0 mm, inner diameter 1.0 mm; Hilgenberg GmbH, Malsfeld, Germany). The initial resistance of the solution-filled pipettes was 0.6–1.4 MΩ. The cGMP-containing solutions were administered via a multi-barrel application system to the cytosolic face of the patch. For studying the activation and deactivation kinetics of CNG channels, we performed fast jumps between different ligand concentrations by means of a double-barreled θ-glass pipette mounted on a piezo-driven device, which was controlled by the computer. For measuring the time courses, the recording rate was 20 kHz. The effective switch-time of the solution exchange, determined with an open-patch pipette and different solutions in the barrels, was negligible compared to the time courses of channel activation and deactivation [38].

### 2.4. cGMP Analogues

All cGMP analogues (Rp-cGMPS (G 016), Rp-8-Br-(2-N)ET-cGMPS (DF156), Rp-(2-N)ET-cGMPS (DF246), and Rp-ß-1,N^2^-Ac-Br-cGMPS (DF235)) were diluted from stock solutions according to the technical details provided by the manufacturer (Mireca Medicines GmbH, Tübingen and Biolog Life Science Institute GmbH & Co. KG, Bremen, Germany). cGMP analogues were prepared according to previously reported methods [30] and US20190292214 (“New equatorially modified polymer linked multimers of guanosine-3′,5′-cyclic monophosphates”). All compounds are >95% pure by HPLC analysis.

### 2.5. Steady-State Concentration–Activation Relationships

Each patch was first exposed to a solution containing no cGMP, and then to a solution containing a saturating cGMP concentration. After subtracting the current in the absence of cGMP, the current-response for each ligand concentration was normalized to the saturating current. The respective concentration–activation relationships were fitted with a Hill equation:

(1) $$\frac{I}{I_{max}}=\frac{1}{1+{\left(\frac{EC_{50}}{x}\right)}^{H}}$$

where *I* is the current amplitude, *I*_max_ is the maximum current induced by a saturating ligand concentration, *x* is the ligand concentration, *EC*_50_ is the ligand concentration of half-maximum effect, and *H* is the Hill coefficient. The concentration–activation relationship describing the inhibitory effect of Rp-8-Br-PET-cGMPS was best fitted by a double Hill equation:

(2) $$\frac{I}{I_{max}}=\frac{a}{1+{\left(\frac{EC_{50,h}}{x}\right)}^{Hh}}+\frac{1-a}{1+{\left(\frac{EC_{50,l}}{x}\right)}^{Hl}}$$

where *I* is the current amplitude, *I*_max_ is the maximum current induced by a saturating ligand concentration, *x* is the ligand concentration, *EC*_50,*h*_ and *EC*_50,*l*_ are the ligand concentrations of half-maximum effect for the high- and the low-affinity component, and *H*_h_ and *H*_l_ are the respective Hill coefficients. *a* and (1 − *a*) represent the amplitudes of the high- and the low-affinity components, respectively.

The activation and deactivation time courses were determined by fitting the respective current traces with single exponentials:

(3) $$I\left(t\right)=A*exp\left[\frac{-t}{\tau }\right]$$

where *A* is the amplitude, *t* the time, and *τ* the time constant for either activation or deactivation.

To be able to differentiate between the effects of the cGMP analogues on the photoreceptor CNG channels, the respective ligands were initially tested on channels that were in a similar activation state (e.g., ~80% activation in the presence of 20 μM cGMP for cone and 100 μM cGMP for rod channels). In this way, we made sure that the observed effects were not the result of cGMP-analogue-dependency on the functional state of the channel. For all experiments, we used 50 µM of the cGMP analogues, because this concentration induced a strong neuroprotective effect with no side-effects in RP-animal models [30].

### 2.6. Tissue Preparation

The mice were kept overnight in a ventilated light-tight box for dark-adaption. Subsequent procedures were performed under dim red light. The eyes of the mice were enucleated; then, the retina was isolated in extracellular solution containing (in mM): 125 NaCl, 26 NaHCO_3_, 2.5 KCl, 2 CaCl_2_, 1 MgCl_2_, 1.25 NaH_2_PO_4_, and 20 glucose, and was maintained at pH 7.4 using carboxygen perfusion (95/5% O_2_/CO_2_). All chemicals were obtained from Merck KGaA (Darmstadt, Germany). For MEA recording, the retinas were placed in the MEA-recording chamber (GC-side down) [39]. During the recordings, the tissue was perfused with carboxygenated medium at 32 °C. The chamber perfusion rate was adjusted to 2 mL/min.

### 2.7. Retinal Recordings

Electrophysiological recordings were performed to assess the light dependent retinal responses (photoreceptors µERG and RGCs spikes) by means of a multi-electrode array system (MEA, USB-MEA60-Up-BC-System-E from Multi Channel Systems, Reutlingen, Germany) equipped with MEA 200/30iR-ITO-pr. The recordings were performed at a 25,000 Hz sampling-rate collecting unfiltered raw data. The trigger-synchronized operation of the light stimulation (LEDD1B T-Cube, Thorlabs) and MEA recording were controlled by the recording protocol set within the MCRack software (v 4.6.2, Multi Channel Systems, Reutlingen, Germany) and the digital I/O box (MCS). The light stimulation (white light LED, 2350 mW, MCWHD3, Thorlabs, Newton, NJ, USA) was applied from beneath the transparent glass MEA, guided by optic fiber and optics. A spectrometer USB4000-UV-VIS-ES (Ocean Optics, Orlando, FL, USA) was employed to calibrate the intensity of the applied light-stimulation protocol. To discriminate the rod and cone photoreceptor responses, a light-stimulation protocol for ex vivo MEA recordings was established following the standard in-vivo ERG protocol [40]. For scotopic recordings (rod responses), the animals were dark-adapted (12 h), while for the photopic recordings (cone responses), the retinal explants were light-adapted on the MEA (5 min at intensity of 4.20 × 10^13^ photons/cm^2^/s), subsequent to scotopic stimulation. The parameters of the light stimuli were set as: light flash 250 ms long (3 repetition per light intensity, 20 s interval). Scotopic: 10 stimuli in 0.5 log steps from −4.5 to 0.0 (1.33 × 10^9^–4.20 × 10^13^ photons/cm^2^/s). Photopic (background light: 4.20 × 10^13^ photons/cm^2^/s): four stimuli in 0.5 log steps from +0.5 to +2.0 (1.33 × 10^14^–4.20 × 10^15^ photons/cm^2^/s). The cGMP analogues were bath-applied and, after a 45 min incubation, light-evoked µERGs as well as RGC spikes were recorded from the same retinal patch.

### 2.8. Data Analysis & Statistics

The statistical analysis of the data from the heterologously expressed CNG channels was performed using the two-tailed unpaired Student’s *t*-test. Experimental data are given as mean ± SEM. Analysis of the experimental data was done with the OriginPro 2016G software (OriginLab Corporation, Northampton, MA, USA).

MEA-recorded raw data files were filtered employing a Butterworth 2nd order filter (MC-Rack, Multi-channel systems) to extract the µERG (field potentials: bandpass 0.01–100 Hz) and spikes (high pass 200 Hz). The filtered data were converted to *.hdf files by MC DataManager (v1.6.1.0, Multi Channel Systems, Reutlingen, Germany) for further data processing in MATLAB [39]. Shown traces data are the average of 30 MEA recording electrodes, per condition. *n* = 5 retinae were recorded per condition. Statistical significance was estimated by one-way ANOVA followed by the Dunnett´s test for multiple comparisons. Experimental data are given as mean ± SEM.

The a-wave slope was calculated as the quotient of the a-wave deflection in µV per 20 ms period (last 20 ms of the a-wave amplitude; between starting point of the a-wave (response latency) and the ending at the a-wave (peak time)).

Figures were prepared using CorelDraw^®^ 2019 (Corel, Ottawa, ON, Canada) and Inkscape (inkscape.org, accessed on 1 February 2021).

## 3. Results

### 3.1. Rp-8-Br-PET-cGMPS Reduces Rod and Cone CNG-Channel Activity

**Figure 1:**
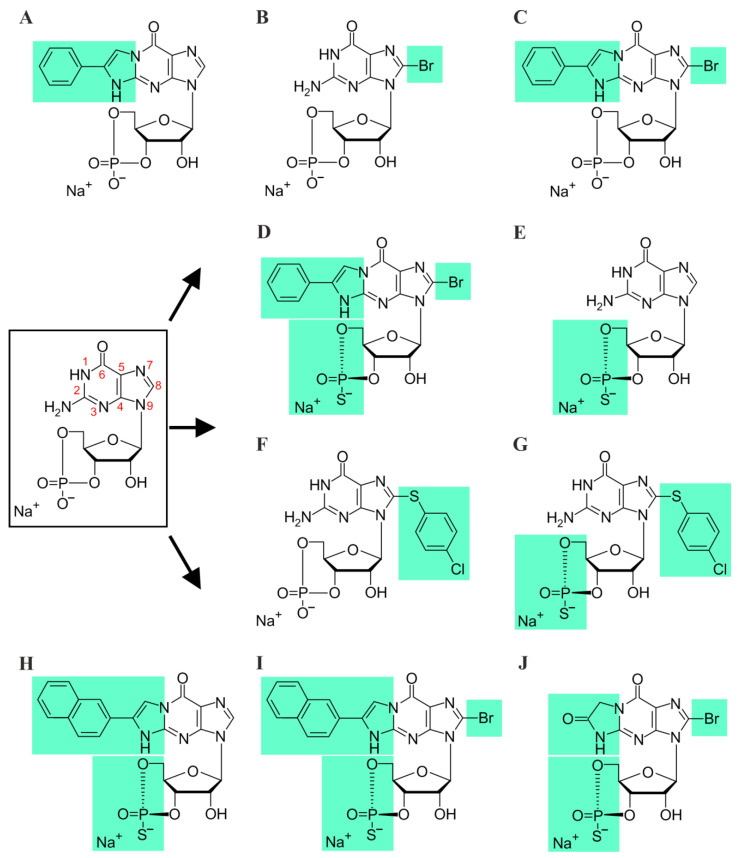
Chemical structures of cGMP and its analogues: (**A**) PET-cGMP, (**B**) 8-Br-cGMP, (**C**) 8-Br-PET-cGMP, (**D**) Rp-8-Br-PET-cGMPS, (**E**) Rp-cGMPS, (**F**) 8-pCPT-cGMP, (**G**) Rp-8-pCPT-cGMPS, (**H**) Rp-(2-N)ET-cGMPS, (**I**) Rp-8-Br-(2-N)ET-cGMPS, and (**J**) Rp-β-1,N^2^-Ac-8-Br-cGMPS. Structure of cGMP is shown in the black box. Green background indicates modifications to the cGMP molecule.

Various cGMP analogues have previously been shown to be efficient modulators of PKG and CNG channels [30,33,34,41]. Here, we tested ten cGMP analogues, including several novel compounds, for their selectivity potential on rod and cone CNG channels (Figure 1). For this, the respective protein isoforms were heterologously expressed in *Xenopus laevis* oocytes. The oocytes co-injected with mRNA coding for CNGA- and CNGB-subunit types expressed heterotetrameric channels composed of CNGA1 and CNGB1a subunits in the case of rods, and of CNGA3 and CNGB3 subunits in the case of cones (Figure S1). These channels exhibited L-*cis*-diltiazem-blocking and cAMP-activation properties similar to native CNG channels [5,7,36,42,43].

**Figure 2:**
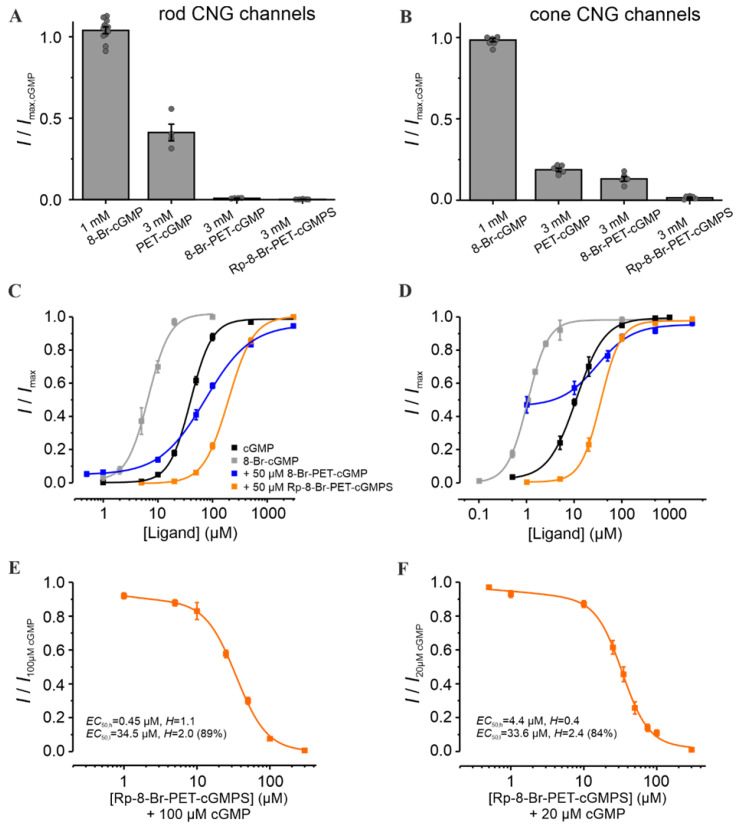
Effect of Rp-8-Br-PET-cGMPS and related cGMP analogues on retinal CNG channels. (**A**,**B**) Efficacy of 8-Br-cGMP, PET-cGMP, 8-Br-PET-cGMP and Rp-8-Br-PET-cGMPS when activating rod and cone CNG channels. The currents measured at +100 mV, in the presence of either 1 mM 8-Br-cGMP or 3 mM of the other cGMP analogues, were related to the maximal current induced by saturating cGMP (3 mM for rod and 1 mM for cone CNG channels; *n* = 4–11). The gray symbols represent individual measurements. (**C**,**D**) Concentration–activation relationships for rod and cone CNG channels in the presence of cGMP (black), 8-Br-cGMP (gray), cGMP + 8-Br-PET-cGMP (50 µM, blue) and cGMP + Rp-8-Br-PET-cGMPS (50 µM, orange). The data points representing means of several experiments were fitted with Equation (1). (**E**,**F**) Rp-8-Br-PET-cGMPS-inhibitory effect on CNG channels in the presence of 100 µM and 20 µM cGMP for rod and cone CNG channels, respectively. The experimental data points representing means of several measurements were fitted with Equation (2) (*n* = 8–16 for rod; *n* = 5–12 for cone; for *EC*_50_, *H,* and n, see Table S1).

For a better understanding of the effect of the Rp-configurated phosphorothioate-modified cGMP analogue Rp-8-Br-PET-cGMPS [30] on retinal CNG channels, we determined first the influence of each of its modifications. cGMP with bromine at position 8 of the guanine-ring system (8-Br-cGMP) had similar efficacy but a higher potency than cGMP when activating the retinal CNG channels (Figure 2A–D). In contrast, cGMP with the spanning β-phenyletheno-modification (PET) at positions N1 and C2 (PET-cGMP) behaved as a partial agonist (Figure 2A,B). Surprisingly, the presence of both modifications in 8-Br-PET-cGMP reduced the efficacy of activating the channels even more (e.g., for rod channel, to less than 1% of maximal activation). A similar effect was observed also for the Rp-8-Br-PET-cGMPS.

To simulate the strong variations of cGMP levels in photoreceptors, especially under RP-like conditions, we measured the effect of the respective cGMP analogues at different cGMP concentrations (Figure 2C,D). Rp-8-Br-PET-cGMPS (50 µM) led to a decrease in the apparent affinity of CNG channels: ~4.9 fold and ~3.2 fold for rods and cones, respectively. Thus, under these conditions, no isoform-selectivity was observed. Interestingly, 8-Br-PET-cGMP had a dual effect: under physiological cGMP it produced a potentiation of retinal CNG-channel activity, whereas at higher cGMP it caused minor inhibition.

To determine the maximal inhibitory potential of Rp-8-Br-PET-cGMPS and to compare its effect on cone and rod CNG channels, additional measurements in the presence of constant cGMP triggering ~80% channel activation (i.e., 100 µM cGMP for rod and 20 µM for cone channel) were recorded (Figure 2E,F). Below 10 µM Rp-8-Br-PET-cGMPS, the concentration of half-maximal inhibition (*EC*_50,h_) for rod channels was ~10 times smaller than that for cones (0.45 µM vs. 4.4 µM). Above 10 µM, the concentration of half-maximal inhibition (*EC*_50,l_) was similar for both CNG-channel types. These results suggest a weak concentration-dependent selectivity of Rp-8-Br-PET-cGMPS for rod over cone CNG channels at low inhibitor concentrations.

### 3.2. 8-pCPT-cGMP Shows a Concentration-Dependent Selectivity for Cone over Rod CNG Channels

**Figure 3:**
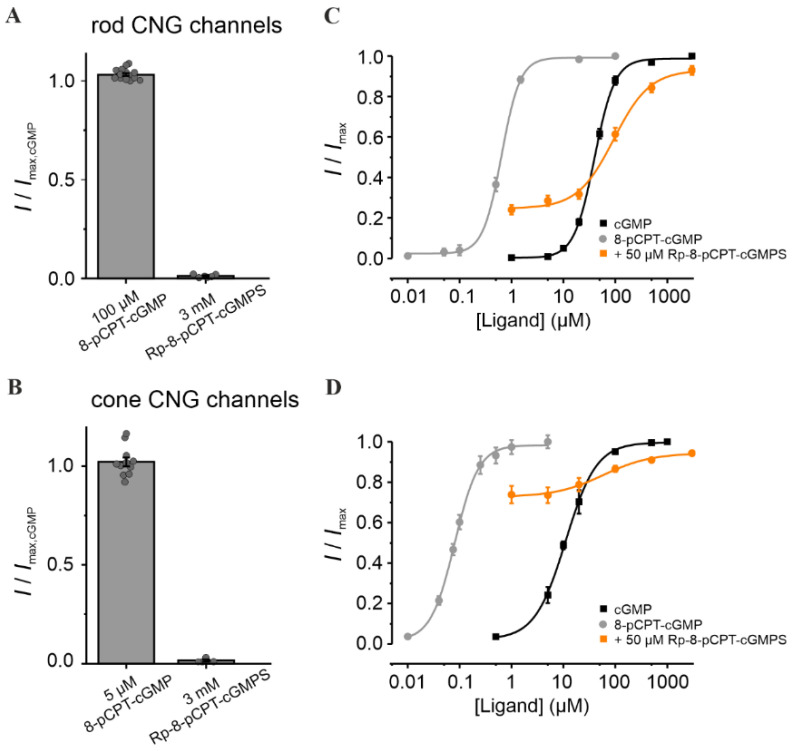
Effect of Rp-8-pCPT-cGMPS and 8-pCPT-cGMP on retinal CNG channels. (**A**,**B**) Efficacy of Rp-8-Br-pCPT-cGMPS and 8-pCPT-cGMP when activating rod and cone CNG channels. The CNG-channel currents triggered by the respective cGMP analogues were related to the maximal cGMP-induced current measured at +100 mV (3 mM and 1 mM cGMP for rod and cone channels, respectively; *n* = 4–16). The gray symbols represent individual measurements. (**C**,**D**) Concentration–activation relationships for rod and cone CNG channels obtained in the presence of cGMP (black), 8-pCPT-cGMP (gray) and cGMP + Rp-8-pCPT-cGMPS (50 µM, orange). The experimental data points, representing means of several measurements were fitted with Equation (1) (for *EC*_50_, *H* and *n* see Table S1).

We next studied the effect of Rp-8-pCPT-cGMPS and of its relative, 8-pCPT-cGMP, i.e., cGMP analogues with a “4-chlorophenylthio”-modification (“pCPT”) at position 8 of the guanine-ring system. Compared to cGMP, 8-pCPT-cGMP had similar efficacy in opening retinal CNG channels, but a much higher potency (~63 times for rod and ~138 times for cone channel; Figure 3 and Table S1). Noteworthy is the robust difference in the apparent affinity of CNG channels for 8-pCPT-cGMP: 0.08 µM for cone vs. 0.63 µM for rod channels. Hence, this compound may enable a selective activation of cone CNG channels. Addition of the “Rp” modification to 8-pCPT-cGMP, in Rp-8-pCPT-cGMPS, drastically reduced the efficacy of this compound to activate both CNG-channel isoforms (Figure 3A,B). These observations differ from those of a previous study where Rp-8-pCPT-cGMPS was estimated to trigger almost ~93% of maximal CNG-channel activation in rods [44]. When the cGMP-activated channels were exposed to 50 µM Rp-8-pCPT-cGMPS, their activity at physiological cGMP increased, and at higher cGMP levels decreased for both channel isoforms. Due to the strong increase in cone-channel activity at physiological cGMP, Rp-8-pCPT-cGMPS was not considered for further studies.

### 3.3. Rp-Modified cGMP Analogues Are Not Selective for Rod or Cone CNG Channels

We then tested four additional Rp-modified cGMP analogues: Rp-cGMPS, Rp-8-Br-(2-N)ET-cGMPS, Rp-(2-N)ET-cGMPS, and Rp-ß-1,N^2^-Ac-Br-cGMPS. These compounds had a lower efficacy than cGMP in opening the retinal CNG channels (Figure S2A,B). Moreover, except for Rp-cGMPS and Rp-ß-1,N^2^-Ac-Br-cGMPS, the cGMP analogues shifted the cGMP-induced concentration–activation relationship of rod and cone CNG channels toward higher cGMP levels (Figure S2C,D). Although in the context of RP-type diseases this effect is desirable for rod channels, the reduction of cone CNG-channel activity at physiological cGMP levels would be detrimental to vision. Rp-cGMPS and Rp-ß-1,N^2^-Ac-Br-cGMPS did not influence the activity of cone channels but, unfortunately, they were also not isoform-selective. When comparing the *EC*_50_-values obtained for all cGMP analogues, we concluded that, none of the compounds had selectivity potential for a specific CNG-channel isoform (Figure S2E,F). Noteworthy is that Rp-8-Br-PET-cGMPS, the cGMP analogue which showed a protective effect in RP-animal models [30], caused the smallest decrease of the channels’ apparent affinity in cones, while the *EC*_50_ value of the rod CNG channel was considerably increased (up to ~5 times; dotted box in Figure S2E,F).

### 3.4. Combination of 8-pCPT-cGMP and Rp-8-Br-PET-cGMPS Preserves Rod and Cone CNG-Channel Function under RP-Like Conditions

**Figure 4:**
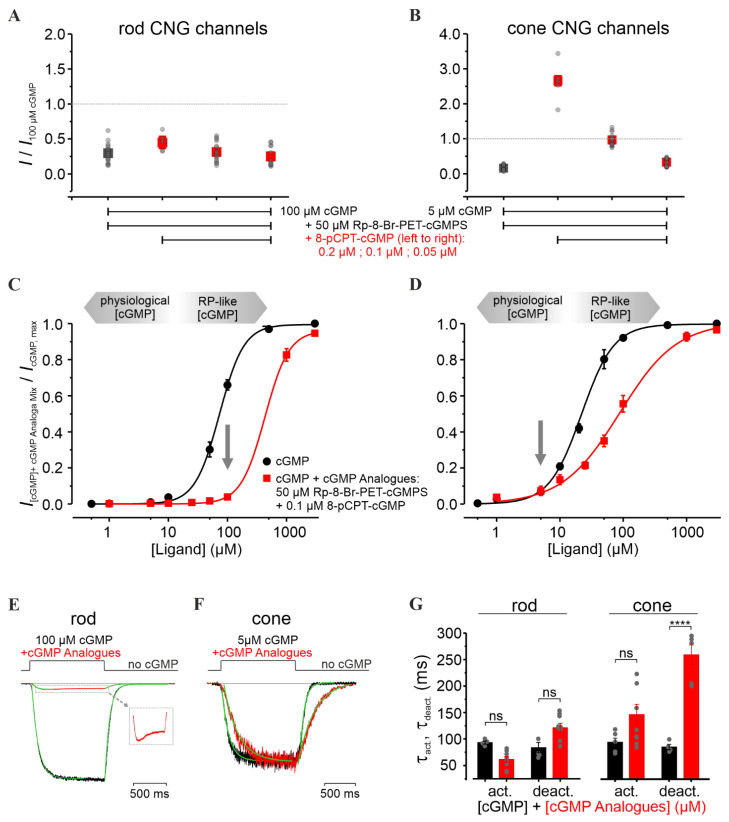
Influence of the combined cGMP-analogues treatment on retinal CNG channels. (**A**,**B**) Relative current amplitudes of rod and cone CNG channels measured under steady-state conditions, in the presence of either cGMP + inhibitor (Rp-8-Br-PET-cGMPS, 50 µM, green) or cGMP + inhibitor (50 µM) + activator (8-pCPT-cGMP, 0.2 µM, 0.1 µM, and 0.05 µM, red). The measured currents were related to the activation triggered by 5 µM and 100 µM cGMP for cone and rod channels, respectively. (**C**,**D**) Concentration–activation relationships for rod (left) and cone (right) CNG channels, measured at −35 mV, in the presence of cGMP (black) and cGMP + inhibitor (50 µM) + activator (0.1 µM) (red). The experimental data points, representing means of several measurements, were fitted with Equation (1) (for *EC*_50_, *H,* and n, see Table S1). cGMP levels < 10 µM were considered physiological conditions, while cGMP levels > 10 µM represent “RP-like”-conditions (gray arrows). (**E**,**F**) Superimposition of representative activation- and deactivation- time courses following a concentration jump from 0 µM cGMP to 100 µM cGMP + 50 µM inhibitor + 0.1 µM activator for rod CNG channels (left) and to 5 µM cGMP + 50 µM inhibitor + 0.1 µM activator for cone (right). The currents triggered by cGMP (black) and by cGMP-analogues co-application (red) were normalized to the level observed in (**C**,**D**). The respective time courses were quantified by a mono-exponential fit yielding the activation and deactivation time constants (τ_act_ and τ_deact_, respectively, Equation (3), green curves). The inset in (**E**) shows the magnified effect of the cGMP-analogues combination on the rod CNG-channel activity (red). (**G**) Activation and deactivation time constants (τ_act_, τ_deact_) for rod and cone CNG channels (*n* = 4–9). Statistical significance (****) was estimated with the Student’s *t*-test. “ns” means not statistically significant. The gray symbols represent individual measurements.

In an attempt to inhibit rod CNG channels, while preserving cone CNG-channel activity, we combined the weak rod-selective inhibitor Rp-8-Br-PET-cGMPS (the “inhibitor”) with the strong cone-selective potent agonist 8-pCPT-cGMP (the “activator”). We studied the CNG-channel activity in the presence of cGMP, inhibitor, and activator, and compared it with that triggered by either cGMP alone, or cGMP and inhibitor only (Figure 4). Several activator concentrations around 0.1 µM (i.e., 0.2 µM and 0.05 µM) were tested, because this concentration triggered ~90% cone CNG-channel activation with almost no effect on rod channels (Figure 3C,D). The selectivity potential of this treatment was estimated at cGMP levels likely to occur in early RP: for cones, at “physiological” cGMP (~5 µM) and for rods, at pathologically high cGMP levels (~100 µM).

Remarkably, the co-application of activator and inhibitor rescued cone-channel activity at physiological cGMP, while inhibiting rod channels at pathological cGMP levels (Figure 4A,B). Up to 5 µM cGMP, the cone-channel activity was very similar to the physiological one, while at 100 µM cGMP, the rod-channel activity was considerably reduced (Figure 4C,D). Moreover, for cone CNG channels, we observed a decrease in the steepness of the concentration–activation relationship (*H* coefficient, Table S1), suggesting that the combined cGMP-analogues treatment reduced the cooperativity during channel gating. This effect was observed only at high cGMP levels, which in RP are not expected to occur in cones [45].

Next, we characterized the effect of the cGMP-analogues combination on the gating kinetics. Although the activation level and the activation-time course (τ_act_) of cone CNG channels under this treatment did not change significantly, the deactivation-time course (τ_deact_) was strongly increased (259.0 ± 18.3 ms vs. 80.6 ± 4.6 ms in the case of cGMP only) (Figure 4F,G). In comparison, rod CNG channels showed almost no change in the gating kinetics (Figure 4E,G). Nevertheless, the fast activation phase of the rod channel was followed by an unexpected decrease in the current amplitude to a constant level (Figure 4E, inset). We speculate that this channel behavior mirrors a faster kinetic effect of the activator compared to that of the inhibitor.

### 3.5. Measuring the Effects of cGMP Analogues on Rod and Cone Light Responses

**Figure 5:**
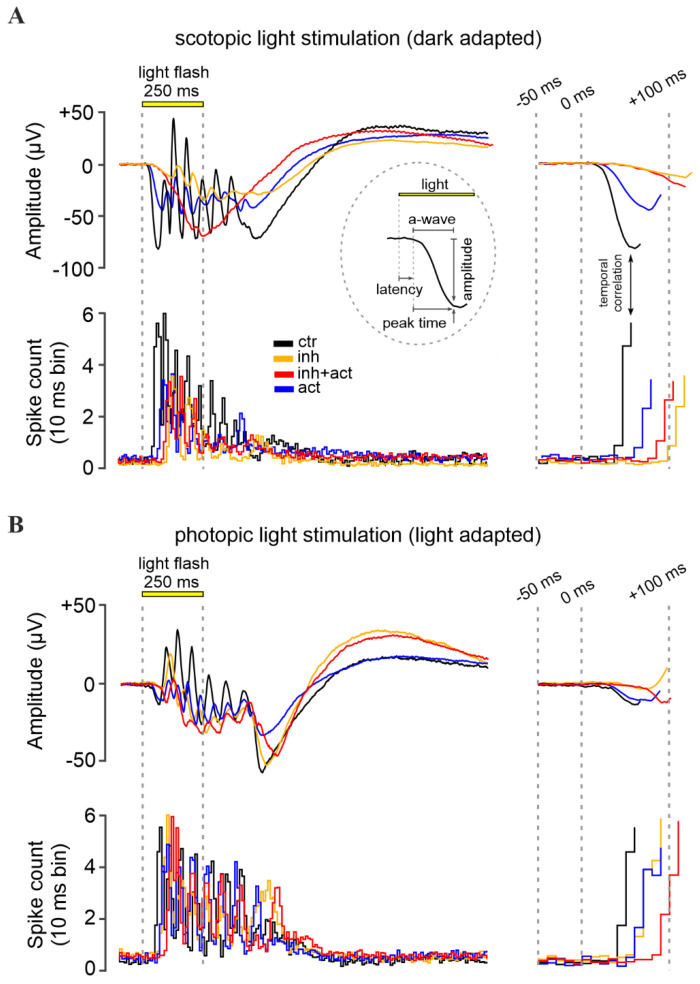
Effects of cGMP analogues on retinal function. Representative light-evoked dark-adapted scotopic (rod) (**A**) and light-adapted photopic (cone) (**B**) micro-electroretinogram (µERG) of the mouse retina. Control recording (black) was compared to retina treated with the CNG-channel inhibitor Rp-8-Br-PET-cGMPS (50 µM, orange), the CNG-channel activator 8-pCPT-cGMP (0.1 µM, blue), or both compounds in combination (red). Displayed below the µERG traces is the correlated retinal ganglion cell spiking activity recorded by multi-electrode arrays (MEA). Data shown at maximum scotopic and photopic light intensity, respectively (see Figures S3 and S4 for µERG recordings at all light intensities). The left panel illustrates the recorded signals, while the right panel highlights the initial light-response features, magnified in time. The initial negative deflection of the µERG, the a-wave, represents the hyperpolarizing photoreceptor response. Subsequent oscillations reflect responses of inner retinal cells. The inset in (**A**) displays the a-wave evaluation parameters: latency, peak-time, and amplitude.

To study the effects of CNG-channel inhibition and/or activation on photoreceptors maintained in their normal histotypic context, we investigated light-evoked electrical responses in mouse retinal explants, utilizing multi-electrode arrays (MEA). Photoreceptor responses were assessed by measuring the negative deflection in the micro-electroretinogram (µERG), which indicates photoreceptor hyperpolarization and corresponds to the so-called a-wave in a conventional human ERG [46] (Figure 5A upper panel and inset). Responses of retinal ganglion cells (RGC) were measured simultaneously (Figure 5A, lower panel). Since RGCs are third-order neurons, their light-correlated response was delayed compared to that of photoreceptors (cf. Figure 5, right). This temporal correlation between a-waves and the onset of RGC spiking activity was used to determine the timing of the a-wave peak. Moreover, to discriminate between rod and cone photoreceptor-specific effects of cGMP analogues, the recordings were carried out under scotopic (dark-adapted, Figure 5A) and photopic (light-adapted, Figure 5B) conditions, respectively.

**Figure 6:**
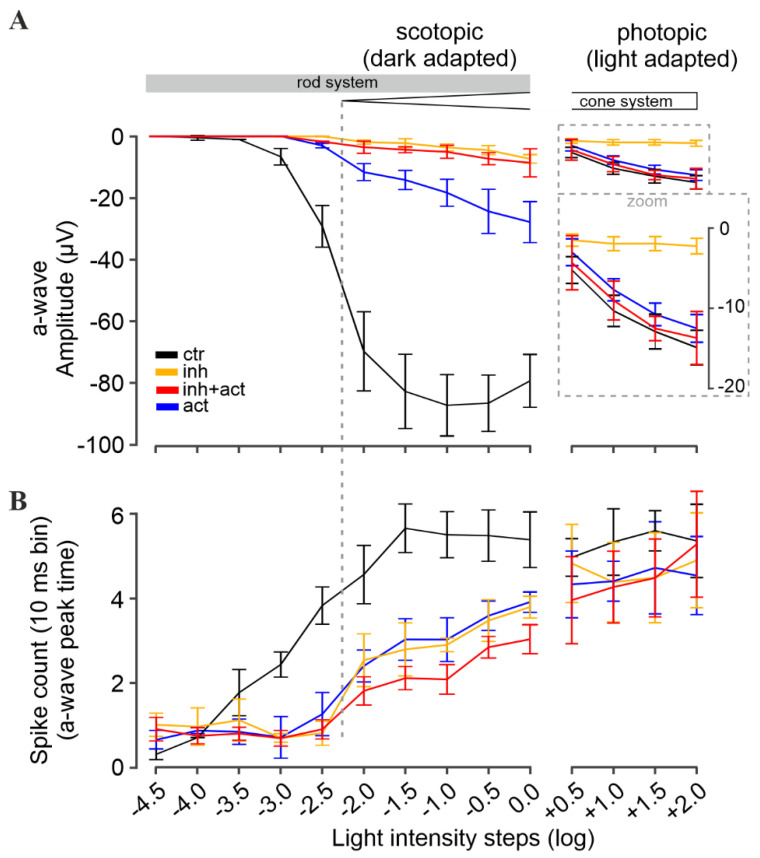
Modulation of retinal light responses by cGMP analogues. Retinal function was measured under scotopic (dark-adapted, left panel) and photopic (light-adapted, right panel) conditions, reflecting rod and cone function, respectively. (**A**) Light-flash-mediated photoreceptor responses shown as a-wave peak amplitudes, and (**B**) correlated retinal ganglion cell spike responses shown as counts per 10 ms bin. Experimental conditions: control (black), inhibitor (Rp-8-Br-PET-cGMPS, 50 µM, orange), activator (8-pCPT-cGMP, 0.1 µM, blue), and inhibitor and activator co-application (red, 50 µM and 0.1 µM, respectively). *n* = 5 retinae for each condition. Data shown averaged from 30 MEA recording electrodes, per condition. See Figures S3 and S4 for the respective µERG trace and spike data, and Tables S2 and S3 for all values and statistical evaluation.

Under scotopic conditions, below −2.0 log units, rod a-wave amplitudes and RGC spiking activity increased with the light intensity in an almost linear fashion (Figure 6A, control, black, see also Figures S3 and S4 for representative traces). At higher light intensities (≥−2.0 log) retinal responses were larger, yet the response intensities of both a-wave and RGC spiking activity eventually saturated. Since ~97% of the mouse photoreceptors are rods while only ~3% are cones [47], the responses recorded within this range were considered as rod-dominant responses. To assess cone responses, the retina was light-adapted to saturate rods so that photopic cone-only responses could be recorded. Due to the low numbers of cones, the photopic a-wave was diminished by ~93%, whereas the RGC spiking responses were comparable to high-light scotopic responses (as the rod pathway relies on the cone–RGC synapses). With increasing light intensities, a slight but continuous increase in the cone a-wave amplitude was observed, while the correlated RGC spike responses appeared to be saturated (Figure 6B).

### 3.6. Rp-8-Br-PET-cGMPS Selectively Silences Rod Photoreceptors

Experiments on heterologously expressed rod and cone CNG channels showed that Rp-8-Br-PET-cGMPS (i.e., the “inhibitor”) reduced their activity. Hence, after pre-incubation of photoreceptors with this compound, fewer CNG channels remained open that could be closed upon light-stimulation, resulting in lower photoreceptor-response amplitudes, mimicking light-adaptation (=saturation).

Accordingly, the incubation of the retina with the inhibitor essentially abolished photoreceptor responses under low scotopic light conditions (<−2.0 log) (Figure 6A, orange). At higher light intensities (≥−2.0 log), a-wave responses were detectable, even though their amplitudes were strongly reduced (~96%). Yet, RGC spiking activity was only moderately reduced (~30–40% drop, Figure 6B, orange). At higher scotopic stimulation, the a-wave and spiking responses were within the range of photopic cone photoreceptor values. This inhibitor-induced behavior resembled the switch from dark-adapted, scotopic stimulation to light-adapted photopic stimulation in the control situation (Figure 6A, black): a strong drop of the a-wave amplitude (~93%), but no significant alterations of spike responses. Hence, it appears that the inhibitor selectively silenced rod responses. In other words, the inhibitor treatment and the subsequent closure of CNG channels emulated a light-dependent saturation of rods, allowing for cone recordings during scotopic stimulation.

In addition, under light-adapted photopic stimulation (≥+0.5 log), a response-dampening effect of the inhibitor on cone photoreceptors was observed (Figure 6A). In control recordings, the a-wave amplitudes increased linearly with increasing light intensities. In contrast, in the presence of the inhibitor, the cone a-wave amplitudes showed only minimal increases. On the other hand, the RGC spiking activity remained nearly at control levels.

Overall, the results indicate that the inhibitor silenced rod photoreceptor light-responses selectively, both in terms of a-wave amplitudes and RGC spiking activity. Importantly, cone a-waves were only dampened by the inhibitor, with cone-dependent RGC activity remaining at control levels.

### 3.7. 8-pCPT-cGMP Counteracts the Inhibitor Effects in Cones but Not in Rods

Next, we investigated the potential of 8-pCPT-cGMP (i.e., the “activator”) to counteract the response-dampening effect of the inhibitor on photoreceptors operating at physiological cGMP levels. In experiments with heterologously expressed rod and cone CNG channels, the activator behaved as a potent agonist and thus created a situation equivalent to dark-adaptation. Interestingly, when the mouse retina was incubated with the activator-and-inhibitor mixture, the activator countered the effect of the inhibitor with a significant preference for cones. The photopic a-wave as well as the spike responses (Figure 6, red) were within the control range, with no significant difference. In the scotopic range, however, the rod-response-dampening effect of the inhibitor remained dominant, even though the activator produced a minor rescue effect: The rod a-wave amplitude increased, and the response was detectable at −2.5 log units, i.e., 0.5 log units earlier in comparison to the inhibitor alone (at −2.0 log). While this improvement of a-wave amplitudes was significant, it was still about ~90% lower than control (>2.5 log). On the other hand, the spiking responses of RGCs were similar to control, especially in the photopic range, again suggesting a cone-selective rescue in the presence of the activator-and-inhibitor mixture.

The incubation of the retina with the activator alone revealed no effect on cones (Figure 6, ≥+0.5 log), but instead produced a marked shift in the sensitivity of rods. Under low scotopic light-stimulation, rod-responses were essentially absent (<−2.0 log range of pure rod-responses, Figure 6A, blue). At higher light intensities (≥−2.0 log), the a-wave amplitudes of the photoreceptors were reduced by 60–80% (Figure 6A), while light-mediated RGC spike responses dropped by only 35–47% (Figure 6B). Moreover, in contrast to control, the response intensities (a-wave and RGC spikes) increased linearly with rising light intensities, suggesting that the rods were not responding at their full capacity (i.e., rods were not saturated). Taken together, our results indicated an activator-mediated shift in rod sensitivity such that higher light intensities were required to elicit responses.

### 3.8. cGMP Analogues Modulate the Kinetics of Photoreceptor Responses

**Figure 7:**
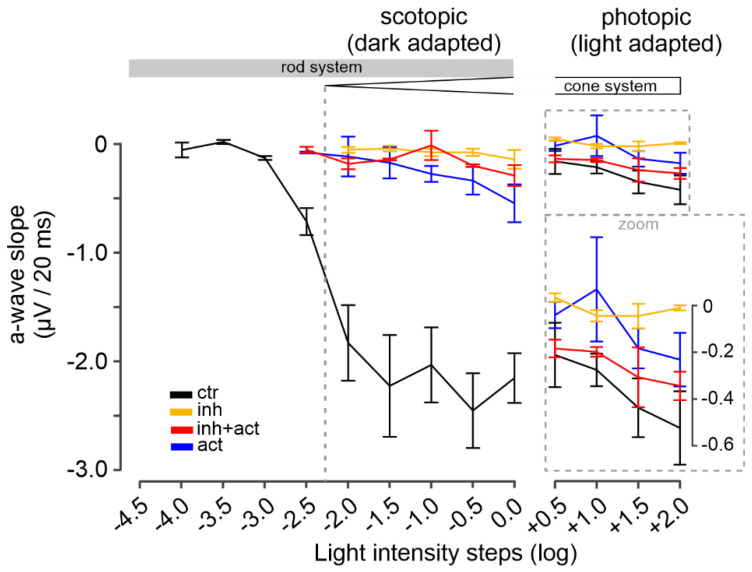
Modulation of photoreceptor-response kinetics by cGMP analogues. Illustration of the a-wave slope calculated as a-wave amplitude deflection in µV per 20 ms. Scotopic (dark-adapted, rod function) light conditions shown in left panel, photopic (light-adapted, cone function) light conditions in right panel. Experimental conditions: control (black), inhibitor (Rp-8-Br-PET-cGMPS, 50 µM, orange), activator (8-pCPT-cGMP, 0.1 µM, blue) and inhibitor-and-activator co-application (red, 50 µM and 0.1 µM, respectively). *n* = 5 retinae for each condition. Data shown are averages of 30 MEA recording electrodes, per condition. See Figures S3 and S4 for the respective µERG trace and spike data, and Tables S4 and S5 for all values and statistical evaluation.

We proceeded further by analyzing the µERG data in the temporal domain, evaluating the a-wave slope (µV per 20 ms) as an indirect measure for the light-intensity- and time-dependent closure of CNG channels. During scotopic stimulation, the control a-wave slope followed the trend of the a-wave amplitudes: they increased linearly with rising light intensities (<−2.0 log, i.e., rod-only range, from 0 to −2.20 ± 0.42 µV/20 ms) and eventually reached saturation at −2.57 ± 0.28 µV/20 ms for light intensities above −2.0 log (Figure 7 left, control, black). In contrast to control, the inhibitor produced a marked (up to 90 %) and significant decrease in the a-wave slope (orange). Since the inhibitor essentially silenced the rod system (cf. Figure 6), the responses obtained within the scotopic range likely relate to the cone system. Remarkably, the co-application of inhibitor and activator (Figure 7, left, red), did not improve the a-wave slope when compared to inhibitor only. On the other hand, the activator treatment alone (blue) decreased the a-wave slope by ~85% in comparison to control; however, this value was still significantly higher than those for the inhibitor (orange) and the inhibitor-and-activator mixture (red).

Under photopic conditions, the control cone a-wave slopes showed a relatively minor increase with light intensity, with no sign of saturation, ranging at an average level of −0.36 ± 0.14 µV/20 ms (Figure 7 right, control, black). For the inhibitor (orange), along with the reduced a-wave amplitude, a prominent decrease in the a-wave slope was also measured (on average 90%). In contrast, the mixture of inhibitor and activator (Figure 7, right, red) produced an 80–90% recovery of the a-wave slope in comparison to control (see Figures S4 and S5 for values and statistical evaluation). The activator alone, however, decreased the a-wave slope by ~60% (averaged) in comparison to control (blue). Overall, the analysis of the photoreceptor-response kinetics supported a cone-selective modulation for the activator and a rod-selective modulation for the inhibitor.

## 4. Discussion

### 4.1. Targeting Rod Photoreceptors

Here, we present a novel approach to not only inhibit rod CNG channels while preserving cone functionality, but also to selectively alter rod and cone photoreceptor visual responses. We employed patch-clamp recordings to characterize direct drug effects on CNG-channel activity and the µERG technique to detect treatment-induced voltage changes in rod and cone photoreceptors maintained in their natural histotypic context. The latter were correlated with retinal ganglion cell spiking activity in order to determine the precise maxima of photoreceptor hyperpolarization.

Despite our best efforts, we could not identify selective inhibitors for rod CNG channels. Even Rp-cGMPS, a known PKG inhibitor previously reported to have opposite effects on rod and olfactory CNG channels [44,48], showed no clear preference for any of the retinal CNG-channel isoforms. All tested compounds either shifted the activation range of both rod and cone CNG channels to higher ligand concentrations, or had no effect at all. Under RP-like cGMP conditions, the co-application of cGMP analogues with antagonistic functional characteristics lead to the inhibition of both rod and cone channels, combined with a selective activation of cone CNG channels only. Thus, primary survival of rods due to CNG-channel inhibition should promote secondary cone survival at functional levels.

This selective modulation of rods was confirmed also on intact photoreceptors operating at natural cGMP levels. While on heterologously expressed CNG channels the inhibitor displayed only a weak selectivity for rod channels, in the retina, the inhibitor essentially silenced rod activity. The activator showed a strong concentration-dependent cone CNG-channel selectivity. The combined cGMP-analogues treatment helped in recovering the cone responses from the inhibitor’s effect, raising additional questions about the modulation mechanism of these compounds.

Under scotopic conditions, the inhibitor decreased the influx of cations by reducing the CNG-channel activity (i.e., mimicking constant light) and shifted the resting potential of both rod and cone photoreceptors to more-negative values. This explains the strongly decreased light-induced hyperpolarization response of the photoreceptors observed in the presence of the inhibitor compared to control. The activator, on the other hand, increased the influx of cations in cone photoreceptors, raising the resting potential to more-positive values (i.e., mimicking constant darkness). In light, this leads to decreased cone-response amplitudes compared to control. Interestingly, the activator under scotopic conditions prevented a response-saturation of rods—even at higher light intensities—indicating a modulation of rod-response kinetics. Our data suggest that under illumination, in the presence of the activator, when cGMP is hydrolyzed, the CNG-channel activity is very briefly reduced, just as long as it takes the activator to reopen them. In other words, the activator forces the photoreceptor into an artificial “dark-adaptation”.

In conclusion, the inhibitor-and-activator combined treatment partially offsets the effect of CNG-channel inhibition in cones, slightly increasing photoreceptor response amplitudes, whereas in rods, it produces a similar effect as the inhibitor alone.

### 4.2. The Combined cGMP-Analogues Treatment Modulates Kinetics of Photoreceptor Responses

Given the highly dynamic nature of our visual environment, the ability of the human eye to promptly register and identify changes is critical for survival. Therefore, the cGMP analogues should elicit their effect without influencing the responsiveness of photoreceptors. Although the inhibitor–activator treatment was intended to target the rod CNG channels only, we observed slower deactivation kinetics of cone channels. The reason for this might be slower ligand unbinding, slower conformational changes within the channel protein upon unbinding, or a combination of both. Surprisingly, at the photoreceptor level, with photopic stimulation and the combined inhibitor–activator treatment, the slope of cone-cell hyperpolarization was barely changed. This suggests, as also previously discussed [49], that the a-wave slope cannot be simply correlated to the kinetics of CNG-channel deactivation only, as it represents a reflection of the photocurrent, i.e., the end-product of the phototransduction cascade. While we also observed a delayed light-response of cone photoreceptors, it is unclear whether this effect would be physiologically relevant. Still, even if adverse changes in kinetics were to be observed, the benefits of preserving vision in RP patients would likely outweigh such side-effects. In addition, confocal patch fluorometry with fluorescently-labeled ligands could be employed to study the binding of cGMP analogues to retinal CNG channels [50]. This could help us to better understand the mechanism of action of these cGMP analogues, and, possibly, to identify and address the changes in the photoreceptor’s responsiveness.

### 4.3. Future Therapy Developments

We have shown that the CNG-channel inhibitor Rp-8-Br-PET-cGMPS, previously reported to delay photoreceptor degeneration in several RP-mouse models [30], when applied alone, was not rod-selective, but also reduced the light-induced responses in cones. To our knowledge, the strategy of combining ligands with agonistic and antagonistic behaviors to achieve channel-selectivity has never been described before, and it is tempting to think of it in terms of a personalized and balanced drug treatment. The tuning of the cGMP analogues’ composition and their respective concentration to selectively control the diseased photoreceptors is a multilevel approach. It requires a basic understanding of their direct effect on cGMP-dependent channels (1), on intact photoreceptors with naturally-balanced cGMP levels (2), and on diseased retinal tissue (3). Our study covered the first two levels of this approach. Future in vivo studies on different RP-animal models may further elucidate the effect of this treatment and its influence on animals’ visual performance.

Nevertheless, challenges may arise from the genetic heterogeneity of different RP forms, different cGMP levels in the diseased cells, and different kinetics of disease development [20,51]. Furthermore, the success of this therapy crucially depends on the time window in which it is applied. Ideally, it should start in the initial phase of RP, when the majority of rod photoreceptors are still physically present, i.e., before the onset of major retinal alterations.

Still to be determined is the effect of this cGMP-analogues combination on other CNG-channel isoforms (e.g., olfactory CNG channels). Unfortunately, there are still many open questions with respect to ligand-selectivity among channel isoforms [5]. Important steps in this direction were made in 2017 when the first full-length structure of a cGMP-bound open-state CNGA1 channel from *C. elegans* was published, and later, from 2020 to 2022, when several rod and cone CNG-channel structures were reported [52,53,54,55,56]. However, it will still be challenging to design selective cGMP analogues since the structure of the ligand-binding domain is highly conserved within the CNG-channel family, making the strategy to use the combined cGMP-analogues treatment even more attractive.

Future studies should also address the effect of such combination treatments on other potential cellular targets containing cGMP-binding domains, such as cyclic nucleotide phosphodiesterases (PDEs) or hyperpolarization-activated, cyclic nucleotide-modulated (HCN) channels. Another key aspect for the success of this treatment will be the kinetics of delivery for the two different cGMP analogues to the photoreceptors. Here, a suitable delivery approach must ensure that both compounds can exert their effects within the same time frame [57].

Our data could also be of strong interest for the treatment of *achromatopsia* and possibly Stargardt disease, rare autosomal recessive cone disorders where the rod-mediated vision remains largely unaffected [58]. Patients suffering from these diseases experience strong photophobia due to the saturation of rod photoreceptors in daylight [59]. Here, a potential therapy could aim at decreasing the sensitivity of rod photoreceptors, such as was shown in this study for the activator treatment. Overall, the implications of our findings may go beyond the treatment of retinal diseases, and could also include the selective regulation of rod and/or cone photoreceptor sensitivity, to improve night or daylight vision, respectively.

## Data Availability

All data are available in the main text or the Supplementary Materials.

## Supplementary Materials

The following supporting information can be downloaded at: https://www.mdpi.com/article/10.3390/pharmaceutics14102102/s1, Figure S1: Electrophysiological characterization of rod and cone heterotetrameric CNG channels expressed in *Xenopus laevis* oocytes; Figure S2: Influence of Rp-modified cGMP analogues on cGMP-induced activation of rod and cone CNG channels.; Figure S3: Effects of cGMP analogues on retinal function under scotopic conditions.; Figure S4: Effects of cGMP analogues on retinal function under photopic conditions; Table S1: Apparent affinity of retinal CNG channels when activated by either cGMP or combinations of cGMP and cGMP analogues; Table S2: Effects of the cGMP analogues on the function of the mouse retina under scotopic and photopic stimulation conditions; Table S3: Statistical evaluation of the effects of the cGMP analogues on the function of the mouse retina under scotopic and photopic stimulation conditions; Table S4: Effects of the cGMP analogues on the temporal response of mouse retina under scotopic and photopic stimulation conditions; Table S5: Statistical evaluation of the effects of the cGMP analogues on the response kinetics of mouse retina under scotopic and photopic stimulation conditions.
