# Supplementary material for: cGMP Analogues with Opposing Actions on CNG Channels Selectively Modulate Rod or Cone Photoreceptor Function
Source: Pharmaceutics. 2022 Oct 1;14(10):2102. doi: 10.3390/pharmaceutics14102102 (PMC9612005; doi:10.3390/pharmaceutics14102102)
Supplement: Supplementary file 1 [file pharmaceutics-14-02102-s001.zip › pharmaceutics-1908850-supplementary.pdf]

Supplementary Information

cGMP analogues with opposing actions on CNG channels selectively modulate rod or cone photoreceptor function

Authors: Sophie Wucherpfennig [1]†, Wadood Haq [2]†, Valerie Popp [1], Sandeep Kesh [1], Soumyaparna Das [3], Christian Melle [4], Andreas Rentsch [5], Frank Schwede [5], François Paquet-Durand [3]*, and Vasilica Nache [1]*

Affiliations:

[1] Institute of Physiology II, University Hospital Jena, Friedrich Schiller University Jena; 07743 Jena, Germany.

[2] Neuroretinal Electrophysiology and Imaging, Institute, Institute for Ophthalmic Research, University of Tübingen; 72076 Tübingen, Germany.

[3] Cell Death Mechanism Group, Institute for Ophthalmic Research, University of Tübingen; 72076 Tübingen, Germany.

[4] Biomolecular Photonics Group, University Hospital Jena, Friedrich Schiller University Jena; 07743 Jena, Germany.

[5] BIOLOG Life Science Institute GmbH & Co KG; 28199 Bremen, Germany.

*Corresponding authors:

Emails: francois.paquet-durand@klinikum.uni-tuebingen.de

and vasilica.nache@med.uni-jena.de

† These authors contributed equally to this work.

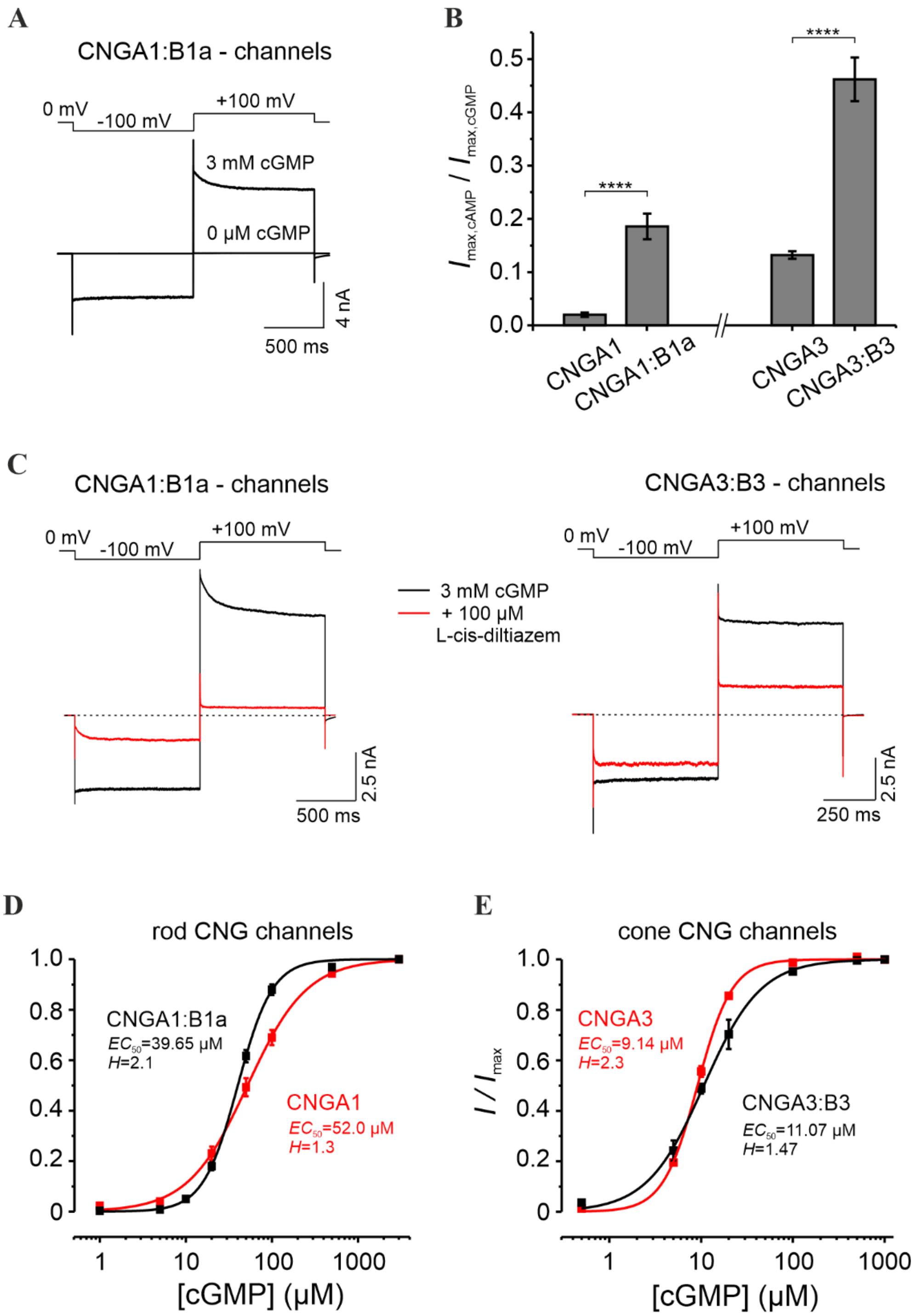

Figure S1. Electrophysiological characterization of rod and cone heterotetrameric CNG channels expressed in *Xenopus laevis* oocytes. **(A)** Representative current traces for rod CNGA1:B1a channels in either the absence or the presence of saturating cGMP. The current traces were elicited by voltage steps from a holding potential of 0 mV to –100 mV, +100 mV and 0 mV. Leak currents

in the absence of cGMP were subtracted from all recordings. (**B)** I_{cAMP}/I_{cGMP} at +100 mV was: 0.02 ± 0.004 for CNGA1 (*n* = 18), 0.19 ± 0.02 for CNGA1:B1a (n = 16), 0.13 ± 0.007 for CNGA3 (*n* = 20) and 0.46 ± 0.04 for CNGA3:B3 channels (*n* = 19). **(C)** Representative cGMP-activated currents from CNGA1:B1a- and CNGA3:B3-channels with (red) or without (black) 100 µM L-*cis*-diltiazem. The voltage protocol is shown at the top. At +100 mV, L-cis-diltiazem blocked ~ 90.5 % of rod- and ~ 63.4 % of cone- CNG channel activity triggered by 3 mM cGMP (*n* = 4). **(D,E)** cGMP-dependent concentration-activation relationships for heterotetrameric rod CNGA1:B1a **(D)** and cone CNGA3:B3 **(E)** channels. The experimental data points, each representing the mean (± SEM) of 5 to 14 measurements, were fitted with Equation (1).

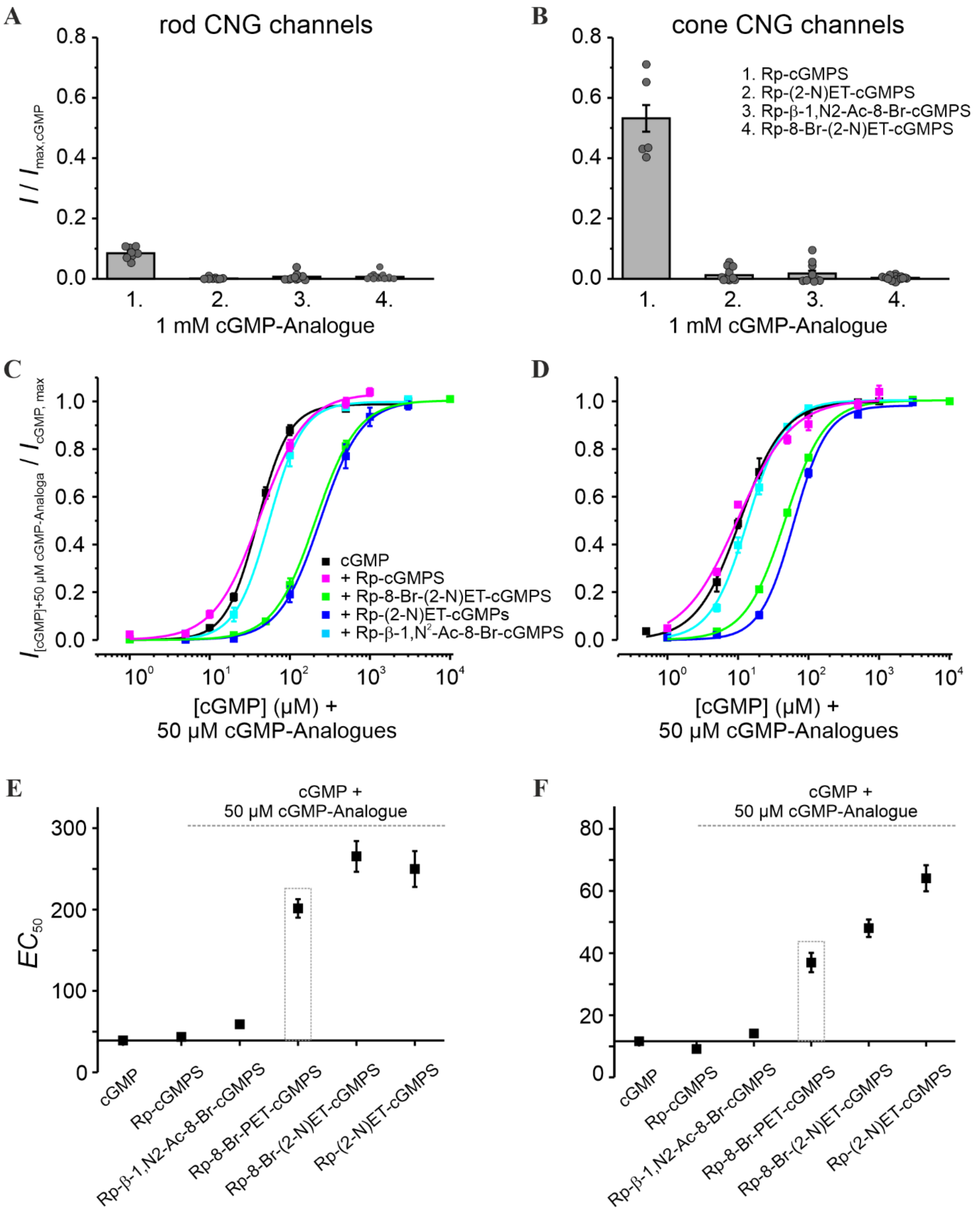

Figure S2. Influence of Rp-modified cGMP analogues on cGMP-induced activation of rod and cone CNG channels. **(A,B)** Efficacy of Rp-8-Br-(2-N)ET-cGMPS, Rp-β-1,N^2-Ac-Br-cGMPS, Rp-cGMPS and Rp-(2-N)ET-cGMPS when activating rod **(A)** and cone CNG channels **(B)**. The currents measured at +100 mV, in the presence of the respective cGMP analogues (1 mM), were

related to the maximal cGMP-current. **(C,D)** Concentration-activation relationships for rod and cone CNG channels, obtained in the presence of cGMP and the respective cGMP analogues. The currents triggered by the cGMP analogues were normalized with respect to the ones obtained at saturating cGMP. The experimental data points, representing means of several measurements, were fitted with Equation (1) (for EC_{50}, H and n see Table S1). **(E,F)** EC_{50} values for rod and cone CNG channels. The dotted box underlines the values obtained for Rp-8-Br-PET-cGMPS.

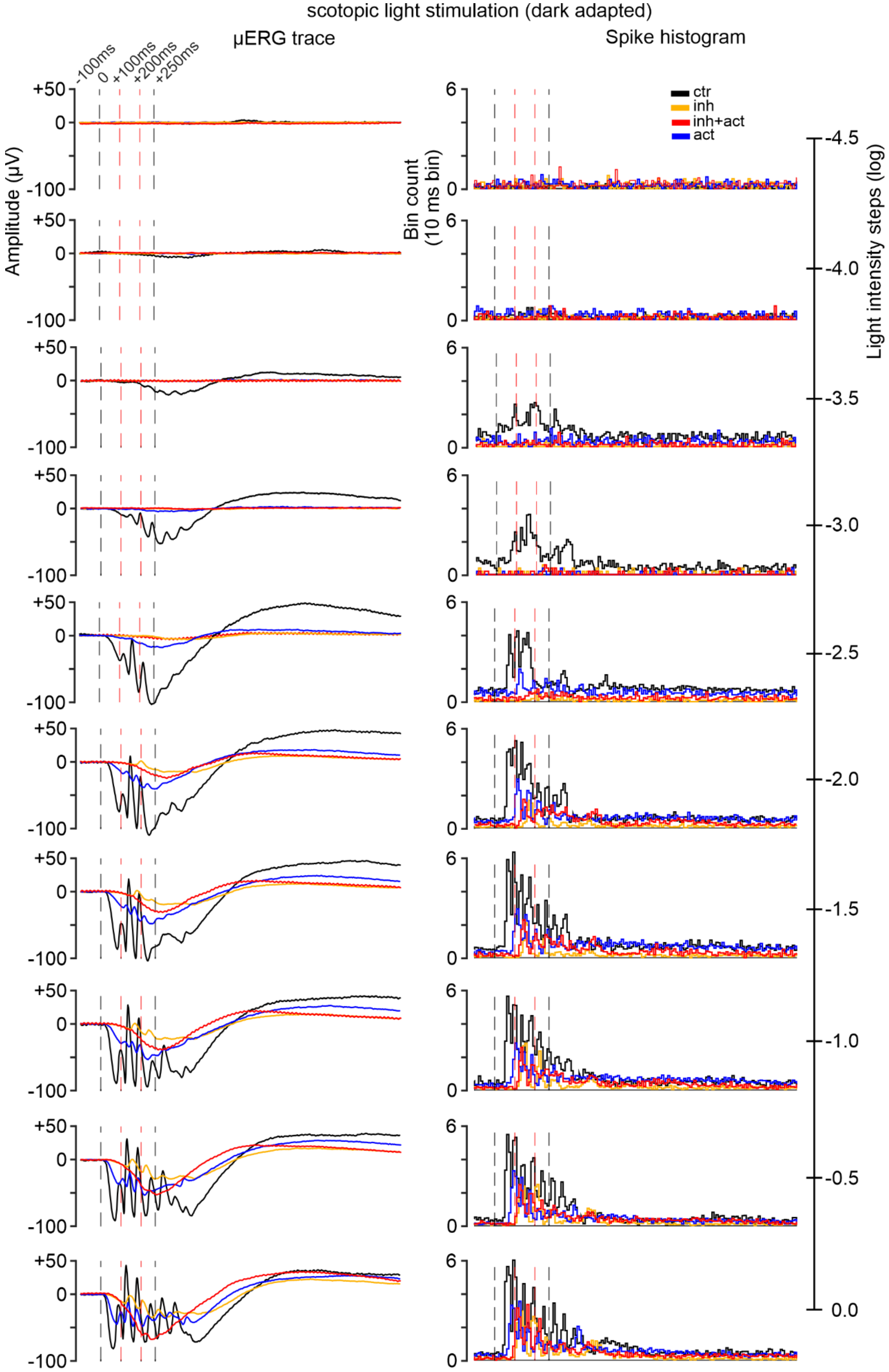

scotopic light stimulation (dark adapted)
µERG trace
Spike histogram
-100ms
0
+100ms
+200ms
+250ms
Amplitude (µV)
+50
0
-100
Bin count (10 ms bin)
6
0
ctr
inh
inh+act
act
Light intensity steps (log)
-4.5
-4.0
-3.5
-3.0
-2.5
-2.0
-1.5
-1.0
-0.5
0.0

Figure S3. Effects of cGMP analogues on retinal function under scotopic conditions**.** Representative electrophysiological recordings featuring **(A)** the µERG traces and **(B)** the corresponding ganglion cells spikes (histogram, 10 ms bin). The summary of this data is presented in Fig. 6. Experimental conditions: control (ctr; black traces), inhibitor (inh; orange), inhibitor and activator combined (inh+act; red) and the activator (act; blue).

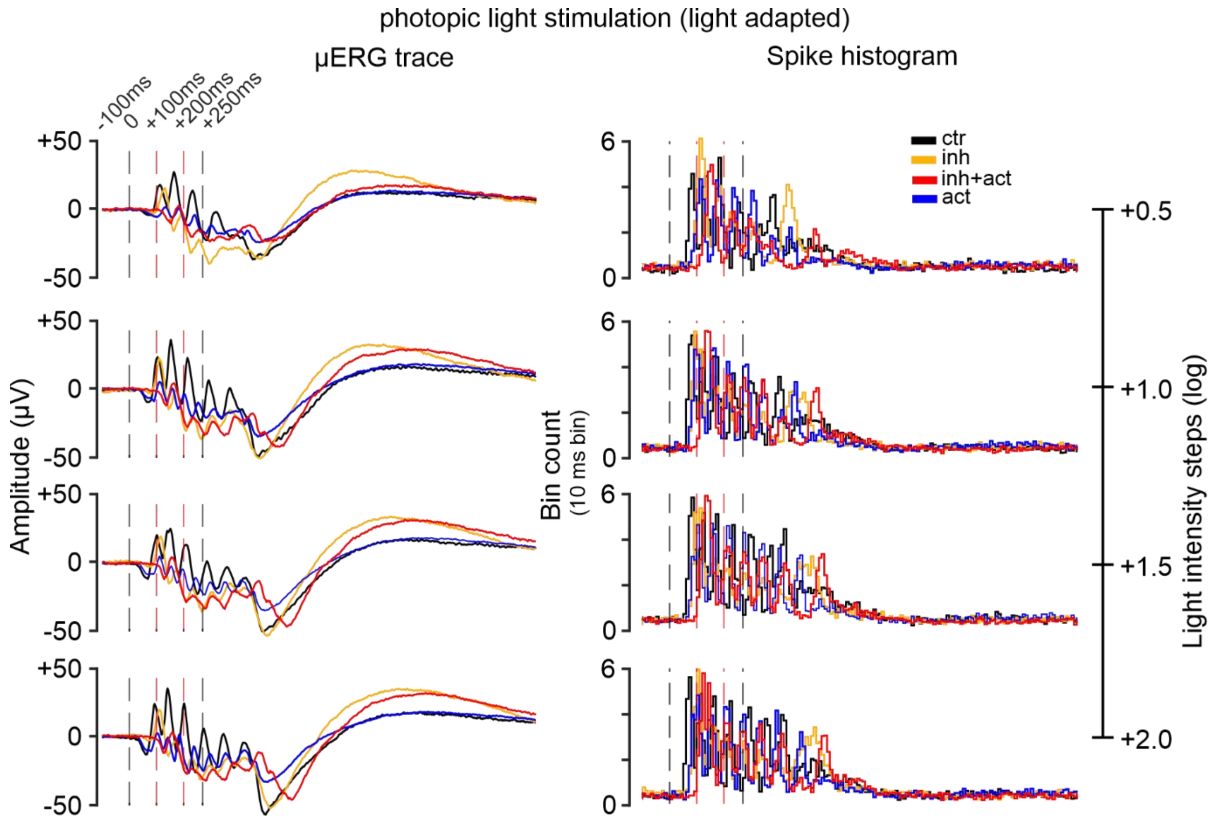

Figure S4. Effects of cGMP analogues on retinal function under photopic conditions. Representative electrophysiological recordings featuring **(A)** the µERG traces and **(B)** the corresponding ganglion cells spikes (histogram, 10 ms bin). The summary of this data is presented in Fig. 6. Experimental conditions: control (ctr; black traces), inhibitor (inh; orange), inhibitor and activator combined (inh+act; red) and the activator (act; blue).

Supplementary Tables

Table S1. Apparent affinity of retinal CNG channels when activated by either cGMP or combinations of cGMP and cGMP analogues. The table shows the EC_{50} values and H (Hill coefficient), obtained from the concentrations-activation relationships presented in Figs. 2-4 and Fig. S2.

**Ligand (µM)**	**Cone CNG channels**			**Rod CNG channels**		
	EC_{50}	***H***	***n***	EC_{50}	***H***	***n***
cGMP	11.07	1.47	6-14	39.65	2.1	4-6
8-Br-cGMP	1.05	1.94	4-7	6.61	2.1	5-11
8-pCPT-cGMP	0.08	1.74	5-12	0.63	2.2	5-16
cGMP + 50 µM 8-Br-PET-cGMP	46.81	1.5	4-6	73.6	1.05	5-9
cGMP + 50 µM Rp-8-Br-PET-cGMPS	35.45	1.97	5	196.4	1.90	6
cGMP + 50 µM Rp-8-pCPT-cGMPS	68.8	1.04	5-7	96.6	1.23	5-6
cGMP + 50 µM Rp-ß-1,N^2-Ac-Br-cGMPS	13.82	1.64	4-8	55.13	2.06	5
cGMP + 50 µM Rp-8-Br-(2-N)ET-cGMPS	46.56	1.53	5-9	250.0	1.66	5-17
cGMP + 50 µM Rp-(2-N)ET-cGMPS	61.78	1.84	4-10	239.9	1.68	5-11
cGMP + 50 µM Rp--cGMPS	9.89	1.13	4-13	42.29	1.52	5-9
cGMP + 50 µM Rp-8-Br-PET-cGMPS + 0.1 µM 8-pCPT-cGMP	87.38	0.96	5-10	429.15	2.1	5-11

Table S2. Effects of the cGMP analogues on the function of the mouse retina under scotopic and photopic stimulation conditions. Presentation of the mean (±std) values for the a-wave amplitude **(A)** and the corresponding spike responses peaking at a-wave **(B)** from the scotopic and photopic stimulation recordings shown in Fig. 6. Experimental conditions: control (ctr), inhibitor (inh), inhibitor and activator combined (inh+act) and the activator (act).

A		Light intensity steps (log)	Experimental condition			
			ctr	inh	inh+act	act
a-wave amplitude (µV)	Scotopic	-4.5	0.00±0.00	0.00±0.00	0.00±0.00	0.00±0.00
		-4.0	-0.49±0.78	0.00±0.00	0.00±0.00	0.00±0.00
		-3.5	-1.00±0.04	0.00±0.00	0.00±0.00	0.00±0.00
		-3.0	-6.61±2.67	0.00±0.00	0.00±0.00	0.04±0.05
		-2.5	-29.18±6.75	0.00±0.00	-1.71±0.13	-3.02±0.74
		-2.0	-69.70±12.83	-1.85±0.71	-3.52±1.96	-11.59±2.75
		-1.5	-82.69±12.05	-2.22±1.40	-4.36±0.96	-14.14±3.10
		-1.0	-87.17±9.94	-3.62±1.17	-5.04±2.10	-18.28±4.38
		-0.5	-86.51±9.14	-4.49±1.49	-7.26±1.94	-24.30±7.17
		0.0	-79.27±8.57	-7.32±1.37	-8.55±4.60	-27.81±6.62
	Photopic	+0.5	-5.26±1.66	-1.53±0.76	-4.36±3.37	-3.04±1.68
		+1.0	-10.36±1.94	-1.98±0.87	-9.06±2.42	-7.72±1.37
		+1.5	-12.93±2.17	-1.96±0.89	-12.55±1.53	-10.79±1.41
		+2.0	-14.92±2.15	-2.27±0.97	-13.74±3.31	-12.55±1.72
**B**		Light intensity steps (log)	ctr	inh	inh+act	act
Spike count (10 ms bin)	Scotopic	-4.5	0.33±0.13	1.03±0.27	0.92±0.28	0.67±0.22
		-4.0	0.73±0.00	0.98±0.44	0.77±0.19	0.89±0.07
		-3.5	1.79±0.55	1.14±0.50	0.82±0.15	0.86±0.30
		-3.0	2.45±0.30	0.72±0.10	0.71±0.19	0.73±0.49
		-2.5	3.84±0.44	0.83±0.28	0.92±0.23	1.28±0.51
		-2.0	4.57±0.69	2.55±0.62	1.83±0.33	2.41±0.38
		-1.5	5.66±0.57	2.80±0.63	2.13±0.27	3.04±0.49
		-1.0	5.51±0.54	2.91±0.16	2.10±0.35	3.03±0.52
		-0.5	5.49±0.60	3.48±0.49	2.86±0.25	3.60±0.35
		0.0	5.39±0.66	3.80±0.26	3.04±0.34	3.92±0.24
	Photopic	+0.5	4.99±0.45	4.85±0.92	3.98±1.03	4.35±0.79
		+1.0	5.36±0.79	4.40±0.94	4.29±0.85	4.43±0.47
		+1.5	5.62±0.48	4.51±1.07	4.50±0.92	4.74±1.09
		+2.0	5.38±0.87	4.92±1.12	5.30±1.25	4.56±0.92

Table S3. Statistical evaluation of the effects of the cGMP analogues on the function of the mouse retina under scotopic and photopic stimulation conditions. Statistical analysis of the scotopic and photopic stimulation recordings shown in Fig. 6 and Table S2: values of the a-wave amplitude **(A)** and the corresponding spike responses peaking at a-wave **(B)**. Statistical significance was estimated by one-way ANOVA followed by the Dunnett´s multiple comparison. Experimental conditions: control (ctr), inhibitor (inh), inhibitor and activator combined (inh+act) and the activator (act).

**A**		Light intensity steps (log)	Comparison of experimental condition					
			ctr<br>inh	ctr<br>act	ctr<br>inh+act	inh<br>act	inh<br>inh+act	act<br>inh+act
a-wave amplitude (µV)	Scotopic	-4.5	1.9e-01 (ns)	5.1e-01 (ns)	1.5e-01 (ns)	6.7e-01 (ns)	1.6e-01 (ns)	7.9e-01 (ns)
		-4.0	7.9e-01 (ns)	3.5e-01 (ns)	6.1e-02 (ns)	1.0e+00 (ns)	2.2e-01 (ns)	5.5e-01 (ns)
		-3.5	6.0e-08 (***)	6.5e-07 (***)	6.6e-10 (***)	8.9e-01 (ns)	9.5e-02 (ns)	7.9e-01 (ns)
		-3.0	2.2e-05 (***)	2.1e-05 (***)	2.1e-06 (***)	1.0e+00 (ns)	2.2e-01 (ns)	2.2e-01 (ns)
		-2.5	2.3e-09 (***)	2.5e-09 (***)	1.4e-09 (***)	1.0e+00 (ns)	8.4e-01 (ns)	6.9e-01 (ns)
		-2.0	7.9e-13 (***)	1.5e-11 (***)	1.5e-12 (***)	1.6e-04 (***)	5.6e-02 (ns)	1.6e-03 (**)
		-1.5	1.2e-14 (***)	2.1e-13 (***)	1.8e-14 (***)	5.0e-04 (***)	2.2e-01 (ns)	7.9e-03 (**)
		-1.0	0.0e+00 (***)	5.6e-15 (***)	0.0e+00 (***)	1.1e-04 (***)	4.2e-01 (ns)	7.9e-04 (***)
		-0.5	3.3e-15 (***)	5.8e-13 (***)	5.6e-15 (***)	1.4e-04 (***)	5.6e-02 (ns)	7.9e-04 (***)
		0.0	5.7e-14 (***)	2.4e-11 (***)	8.0e-14 (***)	1.5e-04 (***)	5.5e-01 (ns)	7.9e-04 (***)
	Photopic	+0.5	7.4e-02 (ns)	6.2e-01 (ns)	1.0e+00 (ns)	9.4e-01 (ns)	9.5e-02 (ns)	6.9e-01 (ns)
		+1.0	5.0e-07 (***)	1.4e-01 (ns)	9.0e-01 (ns)	1.2e-04 (***)	7.9e-03 (**)	5.5e-01 (ns)
		+1.5	8.9e-10 (***)	2.3e-01 (ns)	1.0e+00 (ns)	3.8e-08 (***)	7.9e-03 (**)	9.5e-02 (ns)
		+2.0	3.1e-08 (***)	5.4e-01 (ns)	9.9e-01 (ns)	9.0e-07 (***)	7.9e-03 (**)	5.5e-01 (ns)
**B**		Light intensity	ctr<br>inh	ctr<br>act	ctr<br>inh+act	inh<br>act	inh<br>inh+act	act<br>inh+act

		steps (log)						
Spike count (10 ms bin)	Scotopic	-4.5	1.5e-03 (**)	2.8e-01 (ns)	7.4e-03 (**)	2.5e-01 (ns)	3.3e-01 (ns)	9.5e-02 (ns)
		-4.0	8.1e-01 (ns)	9.9e-01 (ns)	1.0e+00 (ns)	1.0e+00 (ns)	5.2e-01 (ns)	3.3e-01 (ns)
		-3.5	1.8e-01 (ns)	1.8e-02 (*)	1.2e-02 (*)	9.7e-01 (ns)	1.7e-01 (ns)	5.5e-01 (ns)
		-3.0	1.0e-07 (***)	1.2e-07 (***)	9.6e-08 (***)	1.0e+00 (ns)	5.8e-01 (ns)	5.4e-01 (ns)
		-2.5	5.3e-10 (***)	9.2e-09 (***)	9.3e-10 (***)	5.3e-01 (ns)	6.9e-01 (ns)	3.1e-01 (ns)
		-2.0	4.6e-05 (***)	1.9e-05 (***)	5.1e-07 (***)	1.0e+00 (ns)	5.6e-02 (ns)	5.6e-02 (ns)
		-1.5	5.9e-08 (***)	2.4e-07 (***)	1.5e-09 (***)	1.0e+00 (ns)	5.6e-02 (ns)	7.9e-03 (**)
		-1.0	1.5e-08 (***)	3.4e-08 (***)	1.2e-10 (***)	1.0e+00 (ns)	7.9e-03 (**)	1.6e-02 (*)
		-0.5	1.7e-05 (***)	3.7e-05 (***)	2.7e-07 (***)	1.0e+00 (ns)	5.6e-02 (ns)	7.9e-03 (**)
		0.0	1.9e-05 (***)	4.2e-05 (***)	1.5e-05 (***)	1.0e+00 (ns)	7.9e-03 (**)	7.9e-03 (**)
	Photopic	+0.5	1.0e+00 (ns)	8.8e-01 (ns)	3.6e-01 (ns)	9.7e-01 (ns)	3.1e-01 (ns)	6.9e-01 (ns)
		+1.0	4.1e-01 (ns)	4.4e-01 (ns)	2.6e-01 (ns)	1.0e+00 (ns)	8.4e-01 (ns)	6.9e-01 (ns)
		+1.5	4.5e-01 (ns)	7.5e-01 (ns)	4.4e-01 (ns)	1.0e+00 (ns)	8.4e-01 (ns)	6.9e-01 (ns)
		+2.0	1.0e+00 (ns)	8.8e-01 (ns)	1.0e+00 (ns)	1.0e+00 (ns)	6.9e-01 (ns)	2.2e-01 (ns)

Table S4. Effects of the cGMP analogues on the temporal response of mouse retina under scotopic and photopic stimulation conditions. Mean (±std) values of the a-wave slope under scotopic and photopic stimulation recordings shown in Fig. 7. Experimental conditions: control (ctr), inhibitor (inh), inhibitor and activator combined (inh+act) and the activator (act).

		Light intensity steps (log)	ctr	inh	inh+act	act
a-wave slope (µV / 20 ms)	Scotopic	-4.5	0.06±0.02	-0.02±0.03	-0.05±0.02	0.11±0.10
		-4.0	-0.04±0.08	-0.02±0.00	-0.02±0.03	0.02±0.01
		-3.5	0.05±0.02	0.02±0.02	0.13±0.17	-0.04±0.08
		-3.0	-0.13±0.02	-0.04±0.02	-0.02±0.07	0.09±0.15
		-2.5	-0.84±0.15	-0.05±0.03	-0.04±0.03	-0.07±0.01
		-2.0	-2.20±0.42	-0.04±0.03	-0.20±0.06	-0.12±0.22
		-1.5	-2.67±0.57	-0.03±0.03	-0.15±0.01	-0.19±0.17
		-1.0	-2.44±0.42	-0.07±0.04	0.01±0.16	-0.31±0.09
		-0.5	-2.95±0.42	-0.07±0.04	-0.22±0.01	-0.39±0.16
		0.0	-2.59±0.28	-0.15±0.11	-0.33±0.12	-0.64±0.21
	Photopic	+0.5	-0.21±0.14	0.03±0.02	-0.18±0.04	-0.04±0.06
		+1.0	-0.28±0.07	-0.04±0.02	-0.20±0.02	0.07±0.22
		+1.5	-0.44±0.13	-0.05±0.05	-0.31±0.13	-0.18±0.09
		+2.0	-0.52±0.16	-0.01±0.01	-0.34±0.06	-0.23±0.11

Table S5. Statistical evaluation of the effects of the cGMP analogues on the response kinetics of mouse retina under scotopic and photopic stimulation conditions. Statistical significance of the a-wave slope under scotopic and photopic stimulation recordings shown in Fig. 7 and Table S4. The slope was calculated only for a-wave amplitudes exceeding the control base line indicating a retinal light response. Statistical significance was estimated by one-way ANOVA followed by the Dunnett´s multiple comparison. Experimental conditions: control (ctr), inhibitor (inh), inhibitor and activator combined (inh+act) and the activator (act).

		Light intensity steps (log)	ctr	ctr	ctr	inh	inh	act
			inh	act	inh+act	act	inh+act	inh+act
a-wave slope (µV / 20 ms)	Scotopic	-4.5	NaN	NaN	NaN	NaN	NaN	NaN
		-4.0	NaN	NaN	NaN	NaN	NaN	NaN
		-3.5	NaN	NaN	NaN	NaN	NaN	NaN
		-3.0	NaN	NaN	NaN	NaN	NaN	NaN
		-2.5	NaN	2.0e-12 (***)	9.4e-13 (***)	NaN	NaN	9.5e-02 (ns)
		-2.0	8.9e-12 (***)	1.7e-11 (***)	3.6e-11 (***)	1.0e+00 (ns)	7.9e-03 (**)	8.4e-01 (ns)
		-1.5	1.0e-11 (***)	3.1e-11 (***)	2.4e-11 (***)	9.9e-01 (ns)	7.9e-03 (**)	6.9e-01 (ns)
		-1.0	6.4e-13 (***)	4.7e-12 (***)	3.4e-13 (***)	5.6e-01 (ns)	5.5e-01 (ns)	7.9e-03 (**)
		-0.5	8.9e-15 (***)	8.2e-14 (***)	2.4e-14 (***)	1.9e-01 (ns)	7.9e-03 (**)	1.5e-02 (*)
		0.0	1.2e-14 (***)	8.6e-13 (***)	5.2e-14 (***)	2.1e-03 (**)	5.6e-02 (ns)	3.2e-02 (*)
	Photopic	+0.5	1.8e-03 (**)	4.5e-02 (*)	1.0e+00 (ns)	8.6e-01 (ns)	7.9e-03 (**)	7.9e-03 (**)
		+1.0	2.6e-02 (*)	5.2e-04 (***)	9.5e-01 (ns)	6.8e-01 (ns)	7.9e-03 (**)	7.9e-03 (**)
		+1.5	1.5e-05 (***)	3.0e-03 (**)	3.1e-01 (ns)	2.4e-01 (ns)	7.9e-03 (**)	1.5e-01 (ns)
		+2.0	3.8e-07 (***)	8.8e-04 (***)	6.7e-02 (ns)	1.4e-02 (*)	7.9e-03 (**)	2.2e-01 (ns)
